# Genetic Investigation of Inverse Psoriasis

**DOI:** 10.3390/life11070654

**Published:** 2021-07-05

**Authors:** Anikó Göblös, Emese Varga, Katalin Farkas, Kristóf Árvai, Lajos Kemény

**Affiliations:** 1 MTA-SZTE Dermatological Research Group, Eötvös Loránd Research Network, 6720 Szeged, Hungary; lajos.kemeny@hcemm.eu; 2 Department of Dermatology and Allergology, University of Szeged, 6720 Szeged, Hungary; mesi.varga@yahoo.com; 3 Department of Medical Genetics, University of Szeged, 6720 Szeged, Hungary; farkaskatalin88@gmail.com; 4 PentaCore Laboratory, 1094 Budapest, Hungary; kristof.arvai@pentacorelab.hu; 5 HCEMM-USZ Skin Research Group, University of Szeged, 6720 Szeged, Hungary

**Keywords:** inverse psoriasis, genetic background, whole exome sequencing, WES, pathogenic gene variants

## Abstract

Inverse psoriasis is considered to be a rare variant of plaque-type psoriasis and is associated with significantly impaired quality of life. Clinical manifestations and treatment options are somewhat different for each subtype. Identifying genetic variants that contribute to the susceptibility of different types of psoriasis might improve understanding of the etiology of the disease. Since we have no current knowledge about the genetic background of inverse psoriasis, whole exome sequencing was used to comprehensively assess genetic variations in five patients with exclusively inverse lesions. We detected six potentially pathogenic rare (MAF < 0.01) sequence variants that occurred in all investigated patients. The corresponding mutated genes were FN1, FBLN1, MYH7B, MST1R, RHOD, and SCN10A. Several mutations identified in this study are known to cause disease, but roles in psoriasis or other papulosquamous diseases have not previously been reported. Interestingly, potentially causative variants of established psoriasis-susceptibility genes were not identified. These outcomes are in agreement with our hypothesis that the inverse subtype is a different entity from plaque-type psoriasis.

## 1. Introduction

**Figure 1:**
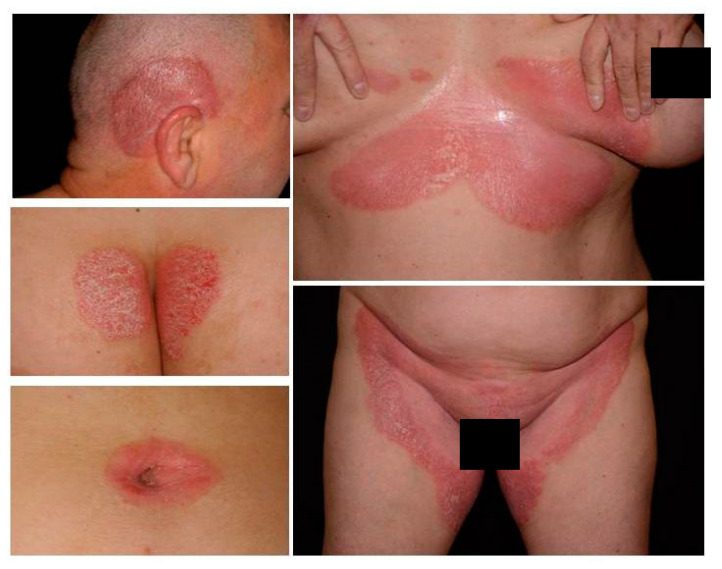
Clinical manifestation of inverse psoriasis.

Psoriasis is a chronic inflammatory skin disorder with a significant occurrence in patients with dermatological diseases. The most common form (90% of all cases) is the plaque-type condition. Inverse psoriasis (flexural, intertriginous), an uncommon variant, is triggered by physical stimuli, infections and obesity. Despite the limited percentage of involved body areas, this variant has a significant adverse impact on quality of life especially regarding sexual behavior [1]. Due to breakthrough development in the therapeutic arsenal, complete or almost complete resolution is usually achieved for most patients with plaque-type psoriasis. Nevertheless, patients with non-plaque phenotypes often do not meet the criteria for these novel agents. As a result, this non-plaque patient population is not adequately assessed in observational and interventional studies and is, therefore, often undertreated [2]. Although up to 63% of psoriasis patients have genital involvement at some point during the course of their disease [3], psoriasis affecting exclusively the genital or flexural regions is very rare. Inverse psoriasis usually affects the skin folds, including the inguinal and axillar fold, genital area, intergluteal cleft, inframammary creases, periumbilical, retroauricular, antecubital and popliteal fossae (Figure 1). The most commonly affected area is the inguinal fold (95.8%), followed by the axillar region and genital area [4]. Morphologically, the lesions often present as well-defined, thin, erythematous plaques without scaling, which can affect both the genital skin and mucosa, and can be accompanied by fissures, ulcers, and/or erosions [5]. Fransson et al. found that patients with palmar psoriasis have increased risk for inverse psoriasis and that the presence of inverse subtypes seems to be linked with periodontitis as well [6]. In challenging cases, dermoscopy or histopathology may be necessary for diagnosis. In vivo confocal reflectance microscopy might also be helpful, although this technology is currently available only in a small number of academic centers due to high equipment costs [7].

The genetic background of psoriasis is complex: more than sixty susceptibility loci have been identified so far, but only a sPSORS (psoriasis susceptibility) loci have been shown to be associated with the disease. Genome-wide association studies (GWAS) and/or genome-wide pathway analysis (GWPA) have shown that more than 200 genes are associated with the disease or the susceptibility to the disease [8,9,10]. Several of these genes implicated in the mechanisms of psoriasis pathology are associated with innate- and adaptive immunity, barrier function, and inflammation.

Although mild cases of inverse psoriasis can be controlled well with topical anti-inflammatory agents, the treatment of more severe symptoms is challenging. The clinical manifestation, location and responses to anti-psoriatic treatments are somewhat different for inverse psoriasis and psoriasis vulgaris, indicating possible genetic diversity for the different subtypes. Nonetheless, a unique genetic profile has not yet been established for inverse psoriasis.

We hypothesized that inverse and non-plaque-type psoriasis are different entities than plaque-type psoriasis rather than rare subtypes of the chronic plaque-type condition. To confirm our hypothesis, we identified gene polymorphisms using whole exome sequencing (WES) analysis.

## 2. Materials and Methods

### 2.1. Patients

Five patients (4 female and 1 male) presenting psoriasis exclusively in inverse areas were recruited from our database. The average age of the patients was 51.4 years. One patient had early-onset psoriasis (onset at 17 years), whereas four patients had late-onset disease (onset at 29–53 years). The affected areas included flexural and genital regions in all of the cases. Four of the patients presented retroauricular involvement, and two patients presented periumbilical involvement, as well.

### 2.2. Blood Samples

Peripheral blood samples were collected from patients diagnosed for inverse psoriasis (*n* = 5) for WES. Variants originating from WES data of other studies were used as a control in-house database. This in-house database included variants of 107 individuals (mean age: 51; without any skin diseases) of Hungarian origin. The investigation was approved by the Ethics Committee of Ministry of Human Capacities, Hungary. Written informed consent was obtained from patients and healthy individuals, and the study was conducted according to the Principles of the Declaration of Helsinki.

### 2.3. DNA Isolation

Genomic DNA was isolated from whole venous blood containing EDTA using the QIAamp DNA Blood Mini Kit (QIAGEN, Hilden, Germany).

### 2.4. Library Preparation and Exome Sequencing

An exome amplicon library was prepared using the Ion AmpliSeq Exome RDY kit (Thermo Fisher, Waltham, MA, USA). Briefly, 100 ng of genomic DNA was added to dehydrated, ultra-high multiplexed primer pairs (12 pools) in a 96-well plate and amplified with the following PCR conditions: 99 °C for 2 min; 99 °C for 15 s and 60 °C for 16 min (10 cycles); and holding at 10 °C. Primers were partially digested using a FuPa reagent, and then sequencing motifs and barcodes were ligated to the amplicons. The library was purified using the Agencourt AMPure XP Reagent (Beckmann Coulter, Brea, CA, USA). The concentration of the final library was determined using the Ion Library TaqMan Quantitation Kit (Thermo Fisher, Waltham, MA, USA) on an ABI 7500 qPCR instrument with the absolute quantification method.

Template preparation was performed with the Ion 540 OT2 Kit (Thermo Fisher, Waltham, MA, USA) on semi-automated Ion OneTouch 2 instrument using the emPCR method. After breaking the emulsion, non-templated beads were removed from the solution during the enrichment process on the Ion OneTouch ES (Thermo Fisher, Waltham, MA, USA) machine. Subsequently, the sequencing primer and polymerase were added, the fully prepared Ion sphere particles were loaded into an Ion 540 chip, and sequencing runs were performed using the Ion S5 Sequencing kit (Thermo Fisher, Waltham, MA, USA) with 500 flows.

### 2.5. Data Analysis

Sequence data from the Ion torrent run were analyzed using the platform-specific pipeline software Torrent Suite, v 5.10 (Thermo Fisher, Waltham, MA, USA), for calling bases, trimming adapter and primer sequences, filtering out poor quality reads, and de-multiplexing the reads according to the barcode sequences. Briefly, the TMAP algorithm was used to align the reads to the hg19 human reference genome, and then the variant caller plug-in was executed to search for germline variants in the targeted regions. The Integrative Genomics Viewer (https://software.broadinstitute.org/software/igv/ accessed on 14 May 2020) was used for visualization of the mapped reads. Variants were annotated using the Ion Reporter software (Thermo Fisher, Waltham, MA, USA).

## 3. Results

Genomic DNA was isolated from peripheral blood samples using a standard technique. WES was performed with samples from five patients who had been definitively diagnosed with inverse psoriasis and were free of common plaque-type psoriasis symptoms. The WES results allowed us to identify 34,800–38,000 sequence variants per person. Many of these variants were common, neutral or synonymous; therefore, we adjusted the filtering criteria as follows: minor allele frequency of less than 0.01; coverage of more than 50; variant functions of missense, nonsense, frame shift or stop loss; and variant present in all five examined inverse psoriasis patients. A total of 186 variants met the filtering criteria and were evaluated by multiple single-nucleotide polymorphism (SNP) databases (Varsome, https://varsome.com accessed on 15 March 2021; Ensembl, https://www.ensembl.org/index.html accessed on 15 March 2021; dbSNP, https://www.ncbi.nlm.nih.gov/snp/ accessed on 15 March 2021) and pathogenicity prediction tools (SIFT, LRT, DANN, PolyPhen, Mutation Taster, FATHMM-MKL, Meta-SVM) to determine whether any could be considered pathogenic. Of 32 mutations (Supplementary Table S1) that were considered to be damaging or probably damaging, six missense variants are located in a functional domain of the corresponding protein (Table 1) and, thus, likely to have consequences at the protein level.

The six possibly causative variants occurred in the following genes: fibronectin (FN1), fibulin (FBLN1), myosin heavy chain 7B (MYH7B), macrophage stimulating 1 receptor (MST1R), ras homolog family member D (RHOD), and sodium voltage-gated channel alpha subunit 10 (SCN10A). None of these genes has been associated with psoriasis in previous large-scale genetic studies (GWAS, GWPA, etc.). All six variants were validated by Sanger sequencing. None of the identified variants were detected in control individuals (*n* = 107, from in-house database).

The rs1250209 missense mutation is located in the fibronectin Type I domain of the fibronectin protein. Fibronectin is a plasma protein that binds cell surfaces and various compounds including collagen, fibrin, heparin, DNA, and actin, and this domain contributes to fibrin binding. Fibronectin is involved in cell adhesion and migration processes, including embryogenesis, wound healing, blood coagulation, host defense, and metastasis. The gene has three regions subject to alternative splicing, resulting in the potential to produce 20 transcript variants. Fibronectin is believed to play a crucial role in the pathogenesis of psoriasis by influencing inflammation and keratinocyte hyperproliferation [11,12,13]. Mutations in fibronectin have been associated with spondylometaphyseal dysplasia [14] and glomerulopathy [15].

Variant rs136730 is located in the anaphylatoxin homologous domain of Fibulin 1. This anaphylatoxin-like domain was identified in the complement-derived anaphylatoxin C3a protein, which mediates smooth muscle contraction, histamine release from mast cells, enhanced vascular permeability, chemotaxis, inflammation, and generation of cytotoxic oxygen radicals [16]. Fibulin 1 localizes to basement membranes, elastic fibers, and other connective tissue structures [17] and is able to bind fibronectin, proteoglycans, tropoelastin, and various elastic fiber and basement membrane proteins [18]. The protein participates in cell adhesion and migration [19] and also contributes to the supramolecular organization of ECM architecture, affecting in particular basement membranes. Fibulin 1 is also a plasma protein capable of binding to fibrinogen [20] and may play a role in hemostasis and thrombosis; moreover, it is implicated in tumor formation and invasion processes as a tumor suppressor [21].

The rs2425015 missense mutation is located in the coiled-coil domain of the MYH7B protein. The *MYH7B* gene encodes the heavy chain of Myosin II, which catalyzes ATP hydrolysis, interacts with actin and is involved in muscle contraction. Several mutations in MYH7 have been associated with inherited cardiomyopathies and muscle atrophy [22,23].

The rs4930409 missense mutation is located on the RHO domain of the RHOD protein. RHOD is involved in endosome dynamics and membrane transport and participates in the reorganization of actin cytoskeleton [24,25]. No pathological mutation of RHOD has been reported in any human disease so far.

Sequence variant rs6599241 is located in the transmembrane domain of the SCN10A protein. The *SCN10A* gene encodes the alpha subunit of a voltage-gated sodium channel which is an integral membrane glycoprotein responsible for the initial rising phase of action in most excitable cells. Sodium channels have been found to accumulate in regions of peripheral nerve injury and may be important in chronic pain [26]. Gain-of-function mutations in SCN10A can cause an episodic pain disorder [27]. In the imiquimod-induced, IL (interleukin)-23-dependent, psoriasis-like skin of mice, SCN10A+ nociceptors interact with DDCs, regulate the IL-23/IL-17 pathway, and control cutaneous immune responses [28].

The rs7433231 missense mutation is located on the tyrosine kinase catalytic domain of the MST1R protein. MST1R is a receptor tyrosine kinase that transduces signals from the extracellular matrix into the cytoplasm by binding to MST1 ligand. This binding regulates many physiological processes, including cell survival, migration and differentiation [29,30]. Dai and coworkers found a significant association between variation in the MST1R gene and development of nasopharyngeal carcinoma [31].

We also filtered the WES results for rare variants of psoriasis susceptibility genes (OMIM database, Singh et al., 2019; Tsoi et al., 2017; Supplementary Table S2). The filtering criteria included a coverage of more than 50, minor allele frequency of less than 0.01, and the variant function. All of the 21 rare variants we identified in individual patients are missense mutations and all were predicted to be benign with SNP pathogenicity prediction tools (Supplementary Table S3). Additionally, we filtered our results for Th17 pathway genes (12 genes, Supplementary Table S4) and identified 58 rare and common SNPs. Although several of these variants have a wide range of variant effect, only two of the sequence variants, each identified in only one patient, were predicted to be possibly damaging (IL-12B, rs3213119, p.Val298Phe and IL-17F, rs2397084, p.Glu126Gly).

## 4. Discussion

We performed WES analysis with samples taken from five adult patients of inverse psoriasis. Careful clinical and histopathological data confirmed the definitive diagnosis inverse psoriasis, and the patients were without common plaque-type psoriasis symptoms. Six of the identified missense mutations are localized in a functional domain of the corresponding protein and were predicted to be pathogenic according to data from SNP databases and computational pathogenicity prediction tools. Notably, none of the identified sequence variants is related to psoriasis-susceptibility genes or genes in pathways associated to psoriasis pathogenesis. Only non-pathogenic variants were found for psoriasis susceptibility genes from the investigated individuals, which agrees well with the absence of classical psoriatic symptoms.

**Figure 2:**
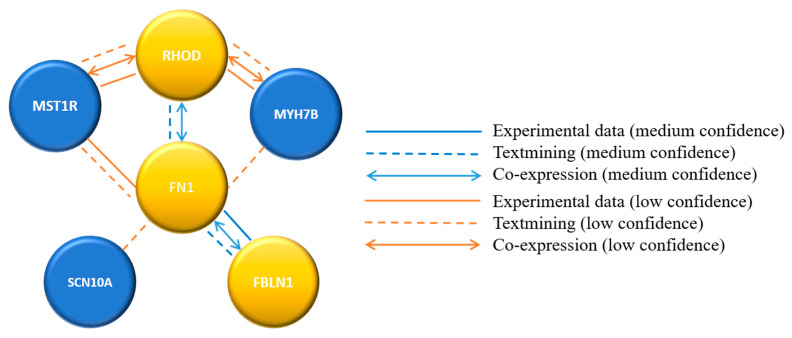
Confidence-based protein-interaction network for variant genes identified in inverse psoriasis.

Sequence variants in six annotated genes presumed to be pathogenic were identified in all five inverse psoriatic patients. These variants were further analyzed for functional protein association networks by the STRING database (STRING, https://string-db.org/ accessed on 23 March 2021). Based on the STRING results, a confidence-based protein-interaction network was calculated for the six identified genes. The analysis predicted medium-confidence association between FBLN1, FN1, and RHOD genes and low-confidence interactions between the other genes (Figure 2).

The miRNAs regulating the six genes were predicted using the GeneCodis tool [32,33,34]. Using a gene set from the inverse psoriasis patients, the co-occurrence annotations found by GeneCodis3 identified the following three miRNAs. miR892a modulates the *FBLN1*, *MYH7B* and *MST1R* genes and is known to promote proliferation and invasion of hepatocellular carcinoma cells via targeting CD226 [35]. miR892b modulates the *FBLN1*, *MYH7B* and *MST1R* genes and is known to influence proliferation, migration, and invasion of bladder cancer cells. In addition, silencing miR892b activates NF-κB in breast cancer [36,37]. miR647 modulates *FBLN1*, *FN1*, and *MST1R* genes and has a tumor-promoting role in gastric cancer via repression of TP73 [38]. This miRNA also promotes cancer progression by downregulating nuclear factor IX in colorectal cancer [39] and has a role in the suppression of human gastric cancer [40].

In conclusion, we detected six potentially pathogenic and rare sequence variants that occurred in all five investigated patients. Notably, none of the potentially causative variants known for psoriasis susceptibility genes were identified, suggesting that the psoriasis of patients with exclusively inverse psoriasis that have lesions only on the skin folds is a different entity from plaque-type psoriasis. It would be important to compare the frequency of the identified variants in inverse and plaque type psoriasis, but unfortunately we did not find any data about these variants in plaque type psoriasis. The major limitation of this study in the low number of examined patients. Although the sequence variants in the six annotated genes were found in all five patients, these findings can not be considered statistically significant due to the small number of enrolled patients. Interestingly our five patients were extremely resistant to antipsoriatic therapies. Topical anti-inflammatory treatments, such as low potent to potent corticosteroids, calcineurin-inhibitors, Vitamin D analogues, did not result in significant improvement of the lesions. Of the conventional therapies, the best results were seen with Cyclosporin A, which was used in three cases; this treatment had to be stopped after two years of use. Three of the patients also received acitretin with no improvement. Methotrexate has been used for all patients with moderate results, and one patient remains on this therapy. After the failures with topical and conventional treatments, biological therapies have been initiated. Biological therapies are antibodies directed to block cytokines responsible for the inflammation associated with psoriasis, such as IL-17 and 23 [41,42]. Treatment using TNF-alfa, IL-12/23, and IL-17 inhibitors had limited efficacy. Two of the five patients are currently receiving IL-23 inhibitors with convincing clearing of the lesions. The results of our genetic investigation and the pattern of treatment resistance of patients suggest that inverse psoriasis has a different pathogenesis than plaque-type psoriasis.

In human genetics, translating genotype data for clinical use is an important step [43]. The biological and clinical interpretation of genetic studies resulted in some therapies already being tested in clinical trials. Understanding how associated variants modulate disease risk and severity, and how they impact cellular phenotypes resulting in more effective drug discovery [44]. Therefore, further large-scale genetic and functional studies are needed to better understand the pathogenesis of this rare form of psoriasis.

## Figures and Tables

**Table 1:** Damaging or probably damaging missense mutations identified in patients with inverse psoriasis.

Variant ID	Location	Gene		Variant Effect	cDNA	Protein	MAF	Transcript	Domain
rs1250209	chr2:216235089	FN1	fibronectin 1	Missense	c.6781G > A	p.Val2261Ile	<0.01	NM_212482.2	Fibronectin type 1
rs136730	chr22:45923827	FBLN1	fibulin 1	Missense	c.422A > G	p.Gln141Arg	<0.01	NM_006486.2	Anaphylatoxin homologous
rs2425015	chr20:33583331	MYH7B	myosin heavy chain 7B	Missense	c.3019A > G	p.Lys1007Glu	<0.01	NM_020884.4	Coiled coil
rs4930409	chr11:66837965	RHOD	ras homolog family member D	Missense	c.400T > C	p.Cys134Arg	<0.01	NM_014578.3	RHO
rs6599241	chr3:38739574	SCN10A	sodium voltage-gated channel alpha subunit 10	Missense	c.5137A > G	p.Met1713Val	<0.01	NM_006514.3	Transmembrane
rs7433231	chr3:49928691	MST1R	macrophage stimulating 1 receptor	Missense	c.3583A > G	p.Ser1195Gly	<0.01	NM_002447.3	Tyrosine kinase, catalytic

## Data Availability

Data is contained within the article or Supplementary Materials.

## Supplementary Materials

The following are available online at https://www.mdpi.com/article/10.3390/life11070654/s1, Supplementary Table S1: damaging or probably damaging sequence variants found in five inverse psoriatic patients; Supplementary Table S2: psoriasis-associated genes based on GWAS and GWPA studies and the OMIM database; Supplementary Table S3: rare variants (MAF < 0.01) located in psoriasis-susceptibility genes from individual inverse psoriasis patients; Supplementary Table S4: rare variants (MAF < 0.01) located in genes involved in the Th17 pathway from individual inverse psoriasis patients.
