# Supplementary material for: Genetic Investigation of Inverse Psoriasis
Source: Life (Basel). 2021 Jul 5;11(7):654. doi: 10.3390/life11070654 (PMC8306123; doi:10.3390/life11070654)
Supplement: Supplementary file 1 [file life-11-00654-s001.zip › life-1257310-SI.pdf]

life

MDPI

Supplementary material

Genetic investigation of inverse psoriasis by Göblös, A et al.

Supplementary Table S1. Damaging or probably damaging sequence variants found in five inverse psoriatic patients.

Variant ID	Gene		Variant Effect	cDNA	Protein	MAF	Transcript
rs10137161	MARK3	microtubule affinity regulating kinase 3	Missense	c.1229T>C	p.Phe410Ser	< 0.01	NM_001128918.2
rs1250209	FN1	fibronectin 1	Missense	c.6781G>A	p.Val2261Ile	< 0.01	NM_212482.2
rs1262686	ADGRG6	adhesion G protein-coupled receptor G6	Missense	c.3380A>G	p.Gln1127Arg	< 0.01	NM_198569.2
rs136730	FBLN1	fibulin 1	Missense	c.422A>G	p.Gln141Arg	< 0.01	NM_006486.2
rs146980577	PRSS3	serine protease 3	Missense	c.333C>G	p.Ser111Arg	< 0.01	NM_007343.3
rs174264	WDSUB1	WD repeat, sterile alpha motif and U-box domain containing 1	Missense	c.518C>T	p.Pro173Leu	< 0.01	NM_001128212.2
rs1774541	HYDIN	axonemal central pair apparatus protein	Missense	c.6259C>T	p.Arg2087Cys	< 0.01	NM_001270974.2
rs204710	SPECC1L, KIAA0376	sperm antigen with calponin homology and coiled-coil domains 1 like	Missense	c.902A>G	p.Asp301Gly	< 0.01	NR_103546.1
rs2058322	AKAP8L	A-kinase anchoring protein 8 like	Missense	c.1374C>G	p.His458Gln	< 0.01	NM_014371.3
rs212146	TPTE	transmembrane phosphatase with tensin homology	Missense	c.1156A>G	p.Lys386Glu	< 0.01	NM_199261.3
rs2425015	MYH7B	myosin heavy chain 7B	Missense	c.3019A>G	p.Lys1007Glu	< 0.01	NM_020884.4
rs2447571	ALDH3B2	aldehyde dehydrogenase 3 family member B2	Missense	c.658A>G	p.Ser220Gly	< 0.01	NM_001031615.1
rs2470103	MYEF2	myelin expression factor 2	Missense	c.1277A>G	p.Gln426Arg	< 0.01	NM_016132.4
rs2512606	KMT5B	lysine methyltransferase 5B	Missense	c.26T>A	p.Ile9Asn	< 0.01	NM_017635.4
rs255051	DPEP2	dipeptidase 2	Missense	c.602C>G	p.Pro201Arg	< 0.01	NM_022355.3
rs2569527	KLK4	kallikrein related peptidase 4	Missense	c.591C>A	p.His197Gln	< 0.01	NM_004917.4
rs2682557	XRCC1	X-ray repair cross complementing 1	Missense	c.1726A>T	p.Asn576Tyr	< 0.01	NM_006297.2
rs28626137	TRIM50	tripartite motif containing 50	Missense	c.37T>C	p.Trp13Arg	< 0.01	NM_178125.3
rs3021119	NRL	neural retina leucine zipper	Missense	c.362A>C	p.Gln121Pro	< 0.01	NM_004563.3
rs3135152	CFAP99	cilia and flagella associated protein 99	Missense	c.1580A>G	p.His527Arg	< 0.01	NM_001193282.2
rs3864938	GCN1	GCN1, eIF2 alpha kinase activator homolog	Missense	c.6463T>G	p.Tyr2155Asp	<0,01	NM_006836.1
rs40470	SOWAHA	sosondowah ankyrin repeat domain family member A	Missense	c.1635T>G	p.Phe545Leu	< 0.01	NM_175873.5
rs4670800	RMDN2	regulator of microtubule dynamics 2	Missense	c.776G>A	p.Gly259Asp	< 0.01	NM_144713.4
rs4803794	CLASRP	CLK4 associating serine/arginine rich protein	Missense	c.520A>G	p.Lys174Glu	< 0.01	NM_007056.2
rs4930409	RHOD	ras homolog family member D	Missense	c.400T>C	p.Cys134Arg	< 0.01	NM_014578.3
rs5927629	TAB3	TGF-beta activated kinase 1 and MAP3K7 binding protein 3	Missense	c.1180T>C	p.Trp394Arg	< 0.01	NM_152787.4
rs6583448	ABCA13	ATP binding cassette subfamily A member 13	Missense	c.13336A>G	p.Lys4446Glu	< 0.01	NM_152701.4
rs6599241	SNC10A, SCNAA	sodium voltage-gated channel alpha subunit 10	Missense	c.5137A>G	p.Met1713Val	< 0.01	NM_006514.3

rs6878669	FAM81B	family with sequence similarity 81 member B	Missense	c.823C>T	p.Pro275Ser	< 0.01	NM_152548.2
rs7433231	MST1R	macrophage stimulating 1 receptor	Missense	c.3583A>G	p.Ser1195Gly	< 0.01	NM_002447.3
rs7637065	MFN1	mitofusin 1	Missense	c.1568C>G	p.Pro523Arg	< 0.01	NM_033540.2
rs908828	ABCA2	ATP binding cassette subfamily A member 2	Missense	c.1751A>C	p.His584Pro	< 0.01	NM_001606.4

Supplementary Table S2. Psoriasis-associated genes based on GWAS and GWPA studies and the OMIM database.

KIAA1109	S100A9	KIAA1107	IL13	BRAP	TAGAP	IL10	MICA	NLRP3
IL2	SLC12A8	CEPT1	IL4	MAPKAPK5	ELMO1	TGFB2	C9	NLRP1
IL21	BSG	DENND2D	STK32A	IL31	DDX58	TNFRSF1A	CFB	RUNX1
IL21-AS1	SMARCA4	PTPN22	TNIP1	UBAC2	KLF4	IL4R	HLA-A	ICAM
BBS12	OR7A10	CYLD	SPATA2	RN7SKP9	ZCH12C	IL1B	HLA-B	FYN
HFE2	TNFRSF9	NOD2	RNF114	RP11-61O1.1	ETS1	LTA	HLA-DMB	ILF3
FLG	TNFRSF1B	FTO	CYP24A1	KLF13	IL23A	IL1A	HLA-DQA2	BAD
LCE3C	KAZN	CDH8	TRAF3IP2	TRIM47	NFKBIA	FLT4	HLA-F	PLAU
LCE3B	IGSF21	SMPD3	IL36RN	TRIM65	SOCS1	CD40	ITGA6	PSMA6
LCE3A	PAX7	CDH3	AP1S3	PTPN2	FBXL19	IL22	C2	SNAI1
LCE3E	CAPZB	IL34	FUBP1	FUT2	NOS2	IL20	CD226	TP63
LCE2C	IFNLR1	MLKL	FASLG	TNFRS9	STAT3	INHBA	HLA-DOB	ESRRA
LCE1C	RUNX3	CMIP	IKBKE	IL-28RA	STAT5A	IL20RA	HSPA1L	SLC44A2
LCE1A	AZIN2	CDH13	ZNF365	LCE3B/C	STAT5B	VEGFA	TAP2	
SMCP	CSMD2	SLC38A8	PTEN	REL	MBD2	IL22RA2	HLADQA2	
IVL	OMA1	MBTPS1	KLLN	B3GNT2	TYK2	HLA-DRA	PRKAR1A	
SPRR2C	IL23R	WFDC1	SNORD74	IFIH1	CARM1	FTO	VWF	
SPRR2G	GNG12-AS1	KIAA0513	CHUK	ERAP1	UBE2L3	STAT2	SDC4	
LELP1	LRRC7	RNF150	CFL1	IL-4	IL1R2	STAT4	ITGA8	

Supplementary Table 3. Rare variants (MAF < 0.01) located in psoriasis-susceptibility genes from individual inverse psoriasis patients.

Variant ID	Location	Gene		Variant Effect	cDNA	Protein	MAF	Transcript
rs41547622	chr6:31238125	HLA-C	major histocompatibility complex, class I, C	missense	c.757G>C	p.Glu253Gln	0.01	NM_001243042.1
rs201429571	chr6:138202173	TNFAIP3	TNF alpha induced protein 3	missense	c.2090G>A	p.Arg697Lys	0.01	NM_001270507.1
rs150375845	chr9:110250334	KLF4	Kruppel like factor 4	missense	c.341C>A	p.Thr114Asn	0.01	NM_004235.5
rs143083792	chr1:15439014	KAZN	kazrin, periplakin interacting protein	missense	c.2140G>T	p.Gly714Cys	0.01	NM_201628.2
rs1136741	chr6:29911901	HLA-A	major histocompatibility complex, class I, A	missense	c.622C>G	p.Pro208Ala	0.01	NM_001242758.1
rs1059563	chr6:29911928	HLA-A	major histocompatibility complex, class I, A	missense	c.649C>G	p.Pro217Ala	0.01	NM_001242758.1
rs201291459	chr6:32714117	HLA-DQA2	major histocompatibility complex, class II, DQ alpha 2	missense	c.714C>G	p.Phe238Leu	0.01	NM_020056.4
rs776170576	chr16:62055238	CDH8	cadherin 8	missense	c.70C>T	p.Pro24Ser	0.01	NM_001796.4
rs79158855	chr18:67563152	CD226	CD226 molecule	missense	c.512G>A	p.Arg171His	0.001	NM_006566.3
rs61757654	chr5:40843550	CARD6	caspase recruitment domain family member 6	missense	c.580A>G	p.Ile194Val	0.003	NM_032587.3
rs146591738	chr1:152276050	FLG	fillagrin	missense	c.11312G>T	p.Gly3771Val	0.01	NM_002016.1
rs141368651	chr1:152276545	FLG	fillagrin	missense	c.10817C>T	p.Ser3606Phe	0.01	NM_002016.1
rs138987068	chr19:10747036	SLC44A2	solute carrier family 44 member 2	missense	c.1271A>G	p.Asn424Ser	0.01	NM_020428.3
rs141554198	chr19:10394455	ICAM1	intercellular adhesion molecule 1	missense	c.630G>T	p.Gln210His	0.01	NM_000201.2
rs114987541	chr3:124826801	SLC12A8	solute carrier family 12 member 8	missense	c.1229G>A	p.Cys410Tyr	0.001	NM_024628.5
rs146865744	chr16:74729601	MLKL	mixed lineage kinase domain like pseudokinase	missense	c.55G>A	p.Glu19Lys	0.001	NM_152649.3
rs34160527	chr1:85020695	SPATA1	spermatogenesis associated 1	missense	c.1145T>C	p.Leu382Pro	0.003	NM_001310156.1
rs146158422	chr1:111702020	CEPT1	choline/ethanolamine phosphotransferase 1	missense	c.358A>G	p.Ile120Val	0.004	NM_001007794.2
rs781595007	chr16:27367155	IL4R	interleukin 4 receptor	missense	c.697C>T	p.Leu233Phe	0.01	NM_001257406.1
rs199734255	chr4:123145669	KIAA1109	KIAA1109	missense	c.2630A>T	p.Asp877Val	0.01	NM_015312.3
rs148589875	chr20:48525003	SPATA2	spermatogenesis associated 2	missense	c.25A>T	p.Thr9Ser	0.01	NM_006038.3

Supplementary Table 4. Rare variants (MAF < 0.01) located in genes involved in the Th17 pathway from individual inverse psoriasis patients.

Variant ID	Gene		MAF	Location	Variant Effect	cDNA	Protein
rs1043730	ACT1/TRAF3IP2	TRAF3 interacting protein 2	0.068	Exonic	Missense	c.969C>A	p.His323Gln
rs878081	AIRE	autoimmune regulator	0.13	Exonic	Synonim	c.588C>T	p.Ser196=
rs1078480			0.46	Intronic	Unknown	c.995+109A>G	-
rs1133779			0.47	Exonic	Synonim	c.1578T>C	p.Asp526=
rs1055311			0.12	Exonic	Synonim	c.681C>T	p.Gly227=
rs41277544			0.025	Intronic	Unknown	c.539-31G>A	-
rs1800521			0.35	Exonic	Synonim	c.1197T>C	p.Ala399=
rs1133779			0.47	Exonic	Synonim	c.1578T>C	p.Asp526=
rs769335225			0.01	Exonic	Synonim	c.1617G>T	p.Pro539=
rs1135314	CARD9	caspase recruitment domain family member 9	0.25	3' UTR variant	Unknown	c.-296T>C	-
rs10781499			0.36	Exonic	Synonim	c.126C>T	p.Pro42=
rs4077515			0.36	Exonic	Missense	c.35G>A	p.Ser12Asn
rs372648269			0.01	Intronic	Unknown	c.627+37G>A	-
rs35051231			0.01	Exonic	Synonim	c.12C>T	p.Tyr4=
-	DECTIN1/DLEC7A	C-type lectin domain containing 7A	-	-	-	-	-
rs3213119	IL12B	interleukin 12B	0.01	Exonic	Missense	c.892G>T	p.Val298Phe
rs919766			0.13	Intronic	Unknown	c.483-36T>G	-
rs3746190	IL12RB1	interleukin 12 receptor subunit beta 1	0.19	3' UTR variant	Unknown	c.2023C>T	-
rs1870063			0.18	Intronic	Unknown	c.1792-67G>A	-
rs401502			0.27	Exonic	Missense	c.1132G>C	p.Gly378Arg
rs375947			0.27	Exonic	Missense	c.1094T>C	p.Met365Thr
rs17852635			0.23	Exonic	Synonim	c.684C>T	p.Pro228=
rs11575934			0.23	Exonic	Missense	c.641A>G	p.Gln214Arg
rs11575926			0.06	Exonic	Missense	c.467G>A	p.Arg156His
rs11086087			0.13	Exonic	Synonim	c.387G>C	p.Val129=
rs436857			0.13	5' UTR variant	Unknown	c.-2C>T	-
rs2397084	IL17F	interleukin 17F	0.03	Exonic	Missense	c.377A>G	p.Glu126Gly
rs917864	IL17RA	interleukin 17 receptor A	0.22	5' UTR variant	Unknown	c.-50T>C	-
rs917865			0.22	5' UTR variant	Unknown	c.-8G>C	-
rs759512999			0.17	Intronic	Unknown	c.932-10C>T	-
rs773943636			0.44	Intronic	Unknown	c.944-28ATCAC>A	-
rs879577			0.27	Exonic	Missense	c.1100C>T	p.Ala367Val
rs879576			0.12	Exonic	Synonim	c.1137G>A	p.Lys379=
rs4819555			0.24	Exonic	Synonim	c.2160C>T	p.Pro720=
rs1468488			0.28	3' UTR variant	Unknown	c.2635T>C	-
rs879575			0.16	Exonic	Synonim	c.1458C>T	p.Ile486=
rs708567	IL17RC		0.39	Exonic	Missense	c.545C>T	p.Ser182Leu
rs279549			0.003	Exonic	Missense	c.1133A>G	p.Gln378Arg
rs279548			0.15	Exonic	Synonim	c.1260C>T	p.Asn420=
rs279545			0.38	Intronic	Unknown	c.1552-86G>A	-
rs183956			0.013	Exonic	Synonim	c.2346C>G	p.Gly782=

rs4686383			0.076	Exonic	Synonim	c.447C>T	p.Thr149=
rs57179462			0.38	Intronic	Unknown	c.1090+28TGG>T	-
rs279549			0.003	Exonic	Missense	c.1133A>G	p.Gln378Arg
rs2066804	STST1	signal transducer and activator of transcription 1	0.22	Intronic	Unknown	c.1874-8C>T	-
rs144704615			0.01	Exonic	Synonim	c.1371G>A	p.Val457=
rs55891000			0.003	Exonic	Synonim	c.1116T>C	p.Asn372=
rs2066800			0.03	Intronic	Unknown	c.634-53A>G	-
rs2066802			0.11	Exonic	Synonim	c.63T>C	p.Leu21=
rs2293152	STAT3	signal transducer and activator of transcription 3	0.35	Intronic	Unknown	c.1233+43C>G	-
rs3830585			0.4	Intronic	Unknown	c.1601-7C>TC	-
rs12720299	TYK2	tyrosine kinase 2	0.09	Intronic	Unknown	c.2311+11G>C	-
rs280519			0.46	Intronic	Unknown	c.1669+7T>C	-
rs280520			0.33	Intronic	Unknown	c.1477-6T>C	-
rs2304256			0.26	Exonic	Missense	c.1084G>T	p.Val362Phe
rs12720270			0.2	Intronic	Unknown	c.1012-36C>T	-
rs34725611			0.27	Intronic	Unknown	c.629+26T>C	-
rs280523			0.08	Exonic	Synonim	c.516C>T	p.Thr172=
